# Comparison of carvedilol versus metoprolol in patients with acute myocardial infarction: A protocol for systematic review and meta-analysis

**DOI:** 10.1097/MD.0000000000025855

**Published:** 2021-05-21

**Authors:** Jian-gang Zhang, Shi-peng Dai, Hua Liu, Ze-sheng Xu

**Affiliations:** Department of Cardiology, Cangzhou Central Hospital, Heibei, China.

**Keywords:** acute myocardial infarction, carvedilol, meta-analysis, metoprolol, protocol, review

## Abstract

**Background:**

The existing meta-analyses and randomized studies on comparing the effects of carvedilol and metoprolol are of poor quality, with small sample sizes, and involve a homogeneous population. Therefore, to provide new evidence-based medical evidence for clinical treatment, we undertook a systematic review and meta-analysis to compare the mortality benefits of carvedilol with metoprolol head to head and determine the better beta-blocker in acute myocardial infarction (AMI) setting.

**Methods:**

Seven electronic databases including Web of Science, Embase, PubMed, Wanfang Data, Scopus, Science Direct, Cochrane Library will be searched in May 2021 by 2 independent reviewers. The protocol was written following the Preferred Reporting Items for Systematic Reviews and Meta-Analyses Protocols (PRISMA-P) statement guidelines. The primary outcome is all-cause mortality; secondary outcomes include complex cardiovascular events, sudden death, cardiovascular death, reinfarction, revascularization, readmission, ventricular arrhythmias, and drug withdrawal for all causes except death. All outcomes are pooled on random-effect model. A *P* value of <.05 is considered to be statistically significant.

**Results:**

The review will add to the existing literature by showing compelling evidence and improved guidance in clinic settings.

**OSF registration number:**

10.17605/OSF.IO/VSTJC.

## 1 Introduction

A growing body of evidence suggests that ventricular repolarization is significantly altered in relation to the development of ventricular arrhythmias during the acute phase of acute myocardial infarction (AMI).^[1]^ In ischemia and reperfusion studies, beta-adrenergic blockers have shown significant antiarrhythmic effects. Although the use of beta-adrenergic blockers in the treatment of AMI has significantly improved clinical outcomes, it is not clear whether they have an effect on cardiac repolarization in the acute phase of AMI.^[2,3]^

Metoprolol is highly selective β_1_-receptor antagonists, whereas carvedilol, in addition to β_1_-receptor blockade, blocks β_2_- and α1-receptors. Metoprolol and carvedilol have previously shown beneficial effects on left ventricular remodeling in patients with heart failure.^[4]^ Placebo-controlled trials of carvedilol and metoprolol resulted in long-term reductions in total mortality, mortality from cardiovascular disease, sudden cardiac death, and death from heart failure.^[5,6]^ Carvedilol or metoprolol, tested in patients with AMI and left ventricular dysfunction, has been shown to reduce left ventricular remodeling, improve left ventricular diastolic filling, and reduce serious cardiac events.^[7]^ However, whether the superiority of carvedilol in patients with AMI remains unclear.

The existing meta-analyses and randomized studies on comparing the effects of carvedilol and metoprolol are of poor quality, with small sample sizes, and involve a homogeneous population.^[8–10]^ Therefore, to provide new evidence-based medical evidence for clinical treatment, we undertook a systematic review and meta-analysis to compare the mortality benefits of carvedilol with metoprolol head to head and determine the better beta-blocker in AMI setting.

## 2 Materials and methods

### 2.1 Protocol registration

The prospective registration has been approved by the Open Science Framework registries, and the registration number is 10.17605/OSF.IO/VSTJC. The protocol was written following the Preferred Reporting Items for Systematic Reviews and Meta-Analyses Protocols (PRISMA-P) statement guidelines

### 2.2 Searching strategy

Seven electronic databases including Web of Science, Embase, PubMed, Wanfang Data, Scopus, Science Direct, and Cochrane Library will be searched in May 2021 by 2 independent reviewers. For search on PubMed, the following search terms will be used: “carvedilol, metoprolol, acute myocardial infarction." To minimize the risk of publication bias, we will conduct a comprehensive search that included strategies to find published and unpublished studies. The reference lists of the included studies will also be checked for additional studies that are not identified with the database search. There is no restriction in the dates of publication or language in the search. No ethical approval is required in our study because all analyses will be based on aggregate data from previously published studies.

### 2.3 Eligibility criteria

Study included in this review has to meet all of the following inclusion criteria in the PICOS order:

1. population: patients with AMI;
2. intervention group (group 1): carvedilol group;
3. comparison group (group 2): metoprolol group;
4. outcome measures: the primary outcome is all-cause mortality; secondary outcomes include complex cardiovascular events, sudden death, cardiovascular death, reinfarction, revascularization, readmission, ventricular arrhythmias, and drug withdrawal for all causes except death;
5. study design: randomized controlled trial.

Biomechanical studies, nonrandomized cohort studies, in vitro studies, review articles, techniques, case reports, letters to the editor, and editorials are excluded.

### 2.4 Study selection

The first author will conduct a preliminary screening based on the title to eliminate any research not related to the topic. A log of excluded studies is kept with the rationale for exclusion. Subsequently, all remaining abstracts will be reviewed by the primary author, and the selection criteria are applied. Studies identified for full text review will be evaluated by 2 authors for inclusion in the study. Disagreements will be resolved through a discussion with a third review author. Journal titles and authors’ names will be not glossed over in the research selection process. A manual search of the bibliographies of included studies is performed to ensure that the overall search was comprehensive and complete.

### 2.5 Data extraction

Two independent authors will extract the following descriptive raw information from the selected studies: study characteristics such as author, study design, study language, publication year, mean follow-up period; patient demographic details such as number, average age, body mass index and gender ratio; details of interventions, and outcome measures. The primary outcome is all-cause mortality; secondary outcomes include complex cardiovascular events, sudden death, cardiovascular death, reinfarction, revascularization, readmission, ventricular arrhythmias, and drug withdrawal for all causes except death. If the data are missing or cannot be extracted directly, we will contact the corresponding authors to ensure that the information integrated. Otherwise, we will calculate them with the guideline of Cochrane Handbook for Systematic Reviews of Interventions 5.1.0. If necessary, we will abandon the extraction of incomplete data.

### 2.6 Statistical analysis

Review Manager software (v 5.4; Cochrane Collaboration) will be used for the meta-analysis. Continuous variables are extracted and analyzed to mean value ± SD. Standardized mean differences with a 95% confidence interval are assessed for continuous outcomes. The heterogeneity is assessed by using the *Q* test and *I*^2^ statistic. An *I*^2^ value of <25% is chosen to represent low heterogeneity and an *I*^2^ value of >75% to indicate high heterogeneity. All outcomes are pooled on random-effect model. A *P* value of <.05 is considered to be statistically significant.

### 2.7 Quality evaluation

The Cochrane risk of bias tool is independently used to evaluate the risk of bias of included randomized controlled trials by 2 reviewers. The quality of randomized controlled trials is assessed by using following 7 items: random sequence generation, allocation concealment, blinding of participants and personnel, blinding of outcome assessment, incomplete outcome data, selective reporting, and other bias. Any controversy is resolved by discussing with a third author to reach a final consensus.

## 3 Discussion

Carvedilol or metoprolol, tested in patients with AMI and left ventricular dysfunction, has been shown to reduce left ventricular remodeling, improve left ventricular diastolic filling, and reduce serious cardiac events.^[7]^ However, whether the superiority of carvedilol in patients with AMI remains unclear. The existing meta-analyses and randomized studies on comparing the effects of carvedilol and metoprolol are of poor quality, with small sample sizes, and involve a homogeneous population.^[8–10]^ Therefore, to provide new evidence-based medical evidence for clinical treatment, we undertook a systematic review and meta-analysis to compare the mortality benefits of carvedilol with metoprolol head to head and determine the better beta-blocker in AMI setting.

## Author contributions

**Conceptualization:** Hua Liu.

**Data curation:** Jian-gang Zhang, Shi-peng Dai.

**Formal analysis:** Jian-gang Zhang, Shi-peng Dai.

**Funding acquisition:** Ze-sheng Xu.

**Investigation:** Jian-gang Zhang, Shi-peng Dai.

**Methodology:** Hua Liu.

**Project administration:** Ze-sheng Xu.

**Resources:** Jian-gang Zhang, Ze-sheng Xu.

**Software:** Hua Liu.

**Supervision:** Ze-sheng Xu.

**Validation:** Shi-peng Dai.

**Visualization:** Hua Liu.

**Writing – original draft:** Jian-gang Zhang.

**Writing – review & editing:** Ze-sheng Xu.
